# Deaths from heart failure: using coarsened exact matching to correct cause-of-death statistics

**DOI:** 10.1186/1478-7954-8-6

**Published:** 2010-04-13

**Authors:** Gretchen A Stevens, Gary King, Kenji Shibuya

**Affiliations:** 1 Information, Evidence and Research, World Health Organization, 20 Avenue Appia, 1211 Geneva, Switzerland; 2 Institute for Quantitative Social Science, Harvard University, 1737 Cambridge Street, Cambridge, MA 02138, USA; 3 Department of Global Health Policy, University of Tokyo, 7-3-1, Hongo, Bunkyo-ku, Tokyo, 113-0033, Japan

## Abstract

**Background:**

Incomplete information on death certificates makes recorded cause-of-death data less useful for public health monitoring and planning. Certifying physicians sometimes list only the mode of death without indicating the underlying disease or diseases that led to the death. Inconsistent cause-of-death assignment among cardiovascular causes of death is of particular concern. This can prevent valid epidemiologic comparisons across countries and over time.

**Methods:**

We propose that coarsened exact matching be used to infer the underlying causes of death where only the mode of death is known. We focus on the case of heart failure in US, Mexican, and Brazilian death records.

**Results:**

Redistribution algorithms derived using this method assign the largest proportion of heart failure deaths to ischemic heart disease in all three countries (53%, 26%, and 22% respectively), with larger proportions assigned to hypertensive heart disease and diabetes in Mexico and Brazil (16% and 23% vs. 7% for hypertensive heart disease, and 13% and 9% vs. 6% for diabetes). Reassigning these heart failure deaths increases the US ischemic heart disease mortality rate by 6%.

**Conclusions:**

The frequency with which physicians list heart failure in the causal chain for various underlying causes of death allows for inference about how physicians use heart failure on the death certificate in different settings. This easy-to-use method has the potential to reduce bias and increase comparability in cause-of-death data, thereby improving the public health utility of death records.

## Background

Effective national and international public health planning and policymaking requires accurate information on population health, especially about deaths and their causes. Death statistics can provide evidence to evaluate health reforms and to identify poorly served populations or diseases. In countries with complete or nearly complete vital registration, including most high-income and some middle-income countries, death statistics are compiled from death certificates. However, inaccurately or incompletely completed death certificates may compromise cause-of-death data in these countries. Physician practice in filling death certificates may vary over place and time [1]. This may result in death rates calculated from death certificate data that are biased or are not comparable across regions, countries, or over time. Inconsistent cause-of-death assignment among cardiovascular causes of death is particularly important, as cardiovascular causes are the leading cause of death, causing 29% of deaths worldwide [2].

Certifying physicians sometimes complete death certificates incorrectly for cardiovascular deaths. Causes such as heart failure and cardiac arrest are routinely used in ways that violate standard protocols. For public health purposes, the underlying cause of death (UCD), as defined by the World Health Organization, should be "the disease or injury which initiated the train of morbid events leading directly to death, or the circumstances of the accident or violence which produced the fatal injury," a definition that is also the most useful for public health monitoring and planning [3]. The UCD listed on the death certificate may be incorrect because of 1) an incorrect diagnosis, or 2) incomplete cause-of-death information. In the second case, the certifying physician often lists only the mode of dying, such as cardiac or respiratory arrest, shock, or heart failure. The World Health Organization's International Statistical Classification of Diseases and Related Health Problems (ICD) specifies that the mode of death should never be designated as the UCD if another plausible cause is listed on the death certificate [3]. Yet certifying physicians regularly list only the mode of dying due to uncertainty about the UCD or lack of knowledge or interest in correct procedures for completing a death certificate [4,5]. Among cardiovascular deaths in the US, 6% are certified to heart failure and 2% to cardiac arrest (Table 1). In Mexico, a middle-income country with a high-quality death registration system, 8% of cardiovascular deaths are assigned to heart failure; in Brazil, 10%. Our goal is to redistribute these deaths into the categories to which they belong.

**Table 1:** Frequency of selected cardiovascular and other causes as the underlying cause of death, US, Mexico, and Brazil.

Cause	ICD-10 codes	US (%)	Mexico (%)	Brazil (%)
***Total number of death records***		***14,500,497***	***920,517***	***3,033,240***

Lower respiratory infections	J10-J18, J20-J22	2.6	3.6	3.6
Cancers	C00-C97	22.9	13.4	13.8
Diabetes	E10-E14	3.0	13.4	3.9
All Cardiovascular diseases	I00-I99	38.1	23.3	27.8
Ischemic heart disease	I20-I25	20.5	10.5	8.4
Cerebrovascular disease	I60-I69	6.7	5.5	8.9
Hypertensive heart disease	I10-I13	2.0	3.0	3.0
Cardiomyopathy	I42-I43	1.1	0.2	1.3
Heart failure	I50	2.3	1.9	2.7
Cardiac arrest	I46, I47.2, I49.0	0.8	0.1	0.1
Other cardiovascular diseases	balance of I00-I99	4.6	2.1	3.3
Chronic obstructive pulmonary disease (COPD)	J40-J44	4.9	3.9	3.4
Digestive diseases	K20-K92	3.5	9.8	4.8
Other diseases		25.0	32.7	42.8

One way to learn how to redistribute these deaths is to compare hospital records or autopsy findings to the cause of death listed on death certificates. Such studies, which have been carried out in the US [6,7] and elsewhere [8,9], often find substantial discrepancies between the death certificate, physician review of hospital records, and autopsy findings. However, these studies are limited by financial and practical constraints. Deaths that occur in hospitals and those selected for autopsy are likely to systematically differ from deaths that do not occur in hospitals and those that are not autopsied. Autopsies, which have been declining in the US and elsewhere [9], may be more likely in difficult-to-diagnose deaths [7], and therefore could be more likely to find less common diseases as the underlying cause of death.

Statistical methods provide an alternative to the autopsy for correcting cause-of-death statistics. Researchers have developed algorithms to redistribute deaths certified to causes that are unspecified or that cannot be underlying causes of death (hereafter referred to as ill-defined causes). Deaths can be reassigned based on expert knowledge of disease etiology, using an empirical basis, or by some combination of the two. A variety of approaches have been taken, including pro-rata redistribution [10], ecological regression analysis [11], and multinomial logistic regression of individual-level data [12]. Aside from pro-rata redistribution, each of these methods requires expert judgment to select the causes to which deaths are redistributed (or target causes of death).

Data compiled from death certificates usually contain both the sequence of conditions that lead to the death and other contributing conditions, called multiple causes of death (MCDs). Unlike the previous approaches, which rely only on underlying cause-of-death data, MCD data allow for an empirical basis to select redistribution targets, and have been used to improve geographic comparability in the use of diabetes as an underlying cause of death using multinomial logistic regression [13]. An empirical redistribution algorithm may result in targets that are not expected based on pathophysiology, but may reflect how modes of death such as heart failure are used in practice.

Though multinomial regression has been used in the past, nonparametric methods are ideal for death certificate data. Multinomial regression requires strong assumptions about how variables are related, which are often violated. It also requires that target causes be broad and distinct (i.e., it limits detailed information about causes to which ill-defined deaths are redistributed). In contrast, nonparametric methods require weaker assumptions and allow for detailed information on target causes. We propose that coarsened exact matching [14], a nonparametric method, be used with MCD data to generate a redistribution algorithm for deaths certified to heart failure (ICD-10 cause I50). The method is demonstrated and validated using death records from two middle-income countries and one high-income country -- Brazil, Mexico, and the US.

Heart failure is a leading ill-defined cardiovascular cause of death in the US and in many other countries [11]. Coronary heart disease is the primary cause of heart failure in the US, but in developing countries, infections such as Chagas disease can play an important role [15]. Hypertension, diabetes, and overweight increase the risk of developing heart failure [16]. Determining heart failure etiology is often complicated by the presence of multiple co-morbid conditions [15].

## Methods

In part 1 of the standard international death certificate, certifying physicians are asked to indicate the sequence of conditions leading directly to the death, listing the UCD last. Part 2 of the death certificate allows the certifier to list other contributing conditions. The underlying cause of death is then selected according to ICD-10 selection rules, typically using linkage tables (in Mexico prior to 2007 and in Brazil) or the automated coding system developed by the US National Center for Health Statistics in the US [17]. Heart failure is only selected as the UCD when no plausible underlying cause is listed in part 1 of the death certificate (ICD rules consider cancers plausible UCDs for this purpose), and neither ischemic heart disease (IHD) nor Chagas disease are listed on the death certificate. Therefore, we treated deaths certified to heart failure as records for which the UCD is missing and drew inferences about possible UCDs for these deaths by using deaths for which heart failure is listed in the causal chain leading to death (i.e., within part 1 of the death certificate). For public health purposes, it is not necessary to assign a unique UCD for each death certified to heart failure. Instead, each death can be distributed among several UCDs, as is the practice in the literature [10-13]. Distributing deaths certified to heart failure among several causes reflects uncertainty about the true UCD. After redistribution, new cause-specific death rates were calculated.

**Figure 1:**
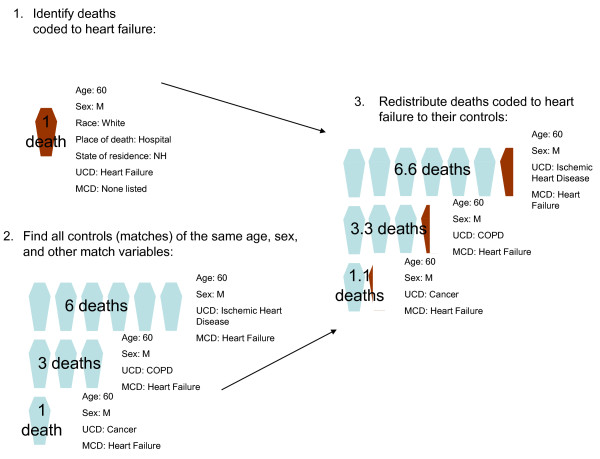
**An example of coarsened exact matching**. UCD: Underlying cause of death; MCD: multiple cause of death; COPD: chronic obstructive pulmonary disease. (1) All deaths with heart failure as the underlying cause of death are identified. In this example, a 60-year-old white male who died in a New Hampshire hospital is identified. (2) All deaths that match the treatment (heart failure) deaths are identified. In this case, all deaths of the same age and sex, with heart failure listed in the causal chain, are identified. (3) The treatment death identified in step 1 is redistributed to the UCDs of the control deaths identified in step 2, proportionally to the number of times each UCD appears among the control deaths. Thus, because 60% of the control deaths identified in step 2 have a UCD of IHD, 60% of the deaths in step 1 are assigned a UCD. The new total of IHD deaths among 60-year-old men is 6.6.

We used coarsened exact matching to generate a distribution of likely causes of death for each death certified to heart failure. Coarsened exact matching is a powerful algorithm but simple to use: the variables on which the match is made are first coarsened (divided into discrete categories), and then all exact matches are made (Figure 1). Thus, each death record certified to heart failure (treatment observations) was matched to all death records where heart failure appeared in part 1 of the death certificate and also had the same value for sex, age, and the other variables listed in Table 2 (control observations). Because the algorithm was not sensitive to how the variables in Table 2 were coarsened, we coarsened the variables until most treatment deaths could be matched to at least one control death. In order to avoid reassigning deaths certified to heart failure to other modes of death or ill-defined causes, we eliminated deaths with these UCDs from the potential control records [18]. In addition, we assumed that physicians would not miscertify injuries to heart failure and eliminated injury deaths from potential matches. Essentially, we matched incomplete death certificates to properly completed death certificates (their controls). Finally, we generated redistribution algorithms by assigning each heart failure death proportionally to the underlying causes of death of all controls. We tested the sensitivity of the method to varying match variables (Table 1), and show two alternate match algorithms:

Demographic specification: match on age, sex, death location, region, and urban/rural, and restrict controls to non-Hispanic whites (US only) with the highest education level and health insurance (Mexico only);

Autopsy specification: match on age, sex, death location, region, urban/rural, and restrict controls to those deaths that were autopsied (autopsy specification).

**Table 2:** Variables on which death records were matched, base specification.

Variable	US	Mexico	Brazil
Age	10-year intervals from age 20 to 49	10-year intervals from age 20 to 49	10-year intervals from age 20 to 49
	5-year intervals from 50-84	5-year intervals from 50-84	5-year intervals from 50-84
	Over 85	Over 85	Over 85
Sex	Male/Female	Male/Female	Male/Female
Death Location	In a clinic or hospital	In a clinic or hospital	In a clinic or hospital
	All other locations	All other locations	All other locations
Region	9 regions	5 regions	5 regions
Urban/rural	Urban/Rural	Urban/Rural	Urban/Rural
Education	Less than high school	Less than primary	None
	At least high school	At least primary	1-7 years
	4-year college or more	Secondary or more	More than 7 years
Race	White		
	Other		
	Black		
Hispanic	Hispanic		
	Non-Hispanic		
Occupation		Professional/technical	
		Informal economy	
Health Insurance System		IMSS	
		Other public or private	
		Seguro popular/none	

IMSS: Mexican Social Security Institute, which provides health insurance to formal-sector workers. Seguro popular: government-subsidized health insurance scheme for the uninsured.

Because congestive heart failure (ICD-10 I50.0) and left ventricular heart failure (I50.1) may be used differently than unspecified heart failure (I50.9), we generated redistribution algorithms considering these causes separately, in addition to generating a redistribution algorithm for all deaths certified to heart failure.

In this paper, the method was applied to individual death records from three datasets: US vital registration death records for the years 1999-2004; Brazilian death records for the years 2003-2005, as provided to the Pan-American Health Organization; and Mexican vital registration data collected by the Health Ministry for 2004-2005 [19]. Mahapatra et al. classified both Mexico and the US as collecting high-quality cause-of-death data, with full coverage and less than 10% use of ill-defined codes [1]. Brazil was classified as collecting medium- to low-quality death statistics due to coverage of approximately 80%, with more than 15% of death records indicating ill-defined causes of death.

## Validation

We also tested the performance of this method by dropping the underlying cause of death for specific groups of US death records that list heart failure among multiple causes of death. We then used matching to predict UCDs. Predicted underlying causes were compared to actual underlying causes using the average relative error (ARE), calculated as follows:

where n is the number of causes considered, *CŜD *is the predicted cause-specific number of deaths, and CSD is the actual cause-specific number of deaths [12]. Because few studies like this one have been carried out, there is little information by which an acceptable ARE can be set a priori. Therefore, it should be considered a descriptive indicator only.

We tested the method in the demographic groups for which heart failure and other ill-defined causes are most frequently used (i.e., where cause-of-death assignment is poor): 1) a region (the Southeastern US, which consists of Alabama, Kentucky, Mississippi, and Kentucky); 2) a racial/ethnic group (all blacks and Hispanics); 3) all deaths on which an autopsy was not performed; and 4) all deaths that occurred out-of-hospital.

The authors had full access to the data and take responsibility for their integrity. All authors have read and agree to the manuscript as written.

## Results

Heart failure (ICD I50) is listed as the underlying cause of death in 2.3% of US death records, 1.9% of Mexican records, and 2.7% of Brazilian records. Of those deaths, 32%, 13%, and 33%, respectively, did not contain any other information on the MCDs; when other causes were listed, they were primarily other ill-defined causes (i.e., cardiac arrest or respiratory failure).

Prior to matching heart failure deaths to other deaths, records with ill-defined or incomplete cause-of-death information -- 7% of total matches -- were eliminated. Ill-defined deaths certified to renal failure (N17-N19), essential hypertension (I10), and general/unspecified atherosclerosis (I70.9) occurred frequently among the potential matches that were eliminated (35%, 5.9%, and 15.5% respectively). A sensitivity analysis was performed, where potential matches were restricted to records with at least three causes listed (i.e., more detailed cause-of-death information was provided), but this had little effect on the results.

In the base analysis, US heart failure deaths were matched to an average of 2,888 death records with mention of heart failure but with another disease as the UCD; Mexican deaths, to 251 records; and Brazilian deaths, to 985 records. Overall, 0.1% of heart failure deaths were not matched to any non-heart-failure death. The aggregate percentage of heart failure deaths redistributed to each underlying cause is shown in Table 3. In all three countries, the largest proportion of heart failure deaths were redistributed to IHD (53%, 26%, and 22% respectively in the US, Mexico, and Brazil). However, a larger proportion of deaths were redistributed to chronic obstructive pulmonary disease, diabetes, and hypertensive heart disease in Mexico and Brazil than in the US. The largest proportion of deaths assigned to cardiomyopathy was in Brazil (9% vs. 4% in the US and 1% in Mexico). In Brazil, an additional 3.7% of heart failure deaths were reassigned to Chagas disease (ICD-10 B57). Because few deaths are certified to Chagas disease, this resulted in a 20% increase in the number of deaths certified to Chagas disease.

**Table 3:** Redistribution algorithm derived under alternate matching algorithms.

	USA			Mexico			Brazil		
	Base (%)	Demographic (%)	Autopsy (%)	Base (%)	Demographic (%)	Autopsy (%)	Base (%)	Demographic (%)	Autopsy (%)
Lower respiratory infections	1	1	2	3	2	2	3	3	1
Diabetes	6	5	2	13	15	12	9	9	3
Cancers	4	4	4	6	8	4	3	3	2
Ischemic heart disease	53	54	54	26	28	33	22	24	41
Cerebrovascular disease	2	2	0	3	3	2	4	4	1
Hypertensive heart disease	7	6	9	16	16	14	23	22	19
Cardiomyopathy	4	4	6	1	1	1	9	9	17
Other cardiovascular diseases	10	11	12	8	8	11	5	6	6
Chronic obstructive pulmonary disease (COPD)	5	5	3	11	8	8	9	8	3
Digestive diseases	1	1	3	4	3	4	2	2	2
Other diseases	7	7	6	9	8	10	11	9	6

Match variables are shown in Table 2. In addition, potential matches were restricted as follows: Demographic: matches (controls) were selected from demographic groups that have the best access to health care (US: non-Hispanic white college graduates; Mexico: secondary school graduates covered by a formal health insurance system; Brazil: individuals with at least seven years of schooling). Autopsy: matches (controls) were selected only from deaths that were autopsied.

Redistribution algorithms were generated by sex, age, and other demographic characteristics. For the US, redistribution algorithms are quite similar across demographic characteristics (See additional file 1: Table S1), with some exceptions for race and ethnicity. Heart failure deaths among blacks were 50% more likely to be redistributed to diabetes; among Hispanics, they were nearly twice as likely. A larger proportion of deaths among blacks was also redistributed to hypertensive heart disease and cardiomyopathy (14% vs. 6% among whites for hypertensive heart disease, and 8% vs. 4% for cardiomyopathy). In Mexico, there was a clear socioeconomic gradient in the proportion of heart failure deaths redistributed to hypertensive heart disease: the proportion was largest among women, deaths occurring outside of a hospital, those with less than primary school completed, and those without health coverage through their employer (Additional file 1: Table S2). A similar pattern was apparent in Brazil (Additional file 1: Table S3).

**Figure 2:**
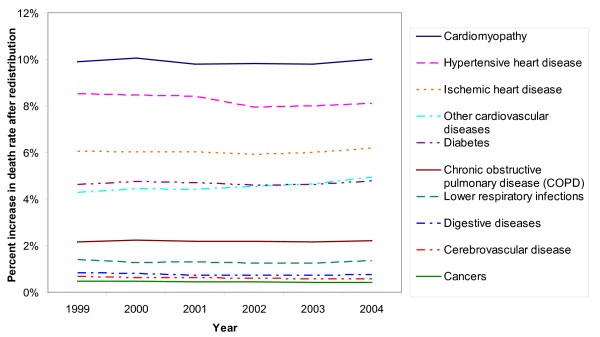
**Increase in US adult death rates after redistribution of heart failure deaths**. Rates are for adults over age 30 and are age-standardized using the US age distribution in the year 2000.

After redistributing heart failure deaths, IHD death rates among US adults over age 30 increased from 3.95 per 1,000 to 4.19 per 1,000; hypertensive heart disease rates increased from 0.38 to 0.41 per 1,000; and cardiomyopathy death rates increased from 0.20 per 1,000 to 0.22 per 1,000. Both absolute and proportional increases in death rates were greater for older age groups, when deaths are more likely to be assigned to heart failure (for example, adjusted IHD death rates for adults over age 85 were 9.5% higher than unadjusted rates vs. 2.3% for adults age 60-64). Changes in death rates varied little between 1999 and 2004 (Figure 2).

Several different specifications of the matching algorithm were tested to determine the effect on the resulting redistribution algorithm (Table 3). For the US data, varying the matching algorithm did not have a major effect on the results. When matched only to deaths for which an autopsy was performed, the percentage of deaths redistributed to digestive diseases and cardiomyopathy increased, and those to diabetes and stroke decreased. However, in these cases, the autopsy results are often not incorporated into the death records, and it is unclear what role selection bias (in terms of the characteristics of deaths that are autopsied) plays.

Results were more sensitive to the specification of the matching algorithm for Mexico and Brazil. In Mexico, when matching to autopsied deaths, the proportion of deaths redistributed to IHD increases (33% vs. 26% in the base specification), and the proportion redistributed to cancers decreases (4% vs. 6%). Likewise, for Brazil, matching only to autopsied deaths results in a substantial increase in the percentage of deaths redistributed to IHD (41% vs. 22%); however, unlike in Mexico, it also doubles the percentage of deaths redistributed to cardiomyopathy (17% vs. 9%). This may reflect more variable epidemiology or quality in cause-of-death assignment in Mexico and Brazil, where patterns in causes of death recorded vary more across population subgroups than in the US.

Congestive heart failure and left ventricular heart failure (ICD-10 I50.0 and I50.1) are used far more frequently than unspecified heart failure (I50.9) in the US, with the pattern reversed in Brazil and Mexico (Additional file 1: Table S4). However, specified heart failures (congestive and left ventricular) were associated with the same underlying causes as unspecified heart failure, and the redistribution algorithm varied little by heart failure type in all three countries.

When tested by dropping underlying cause-of-death information for specific population subgroups in the US, the method performed well (ARE of 19% when underlying causes of death in Southeastern states were predicted; ARE of 22% when causes for all non-white deaths were predicted). However, when the method was used to predict the cause-of-death distribution for all out-of-hospital deaths and for all nonautopsied deaths, it performed less well (ARE of 31% and 35%, respectively).

## Discussion

In this paper, we proposed using coarsened exact matching to predict the likely UCD when heart failure was assigned as the UCD on death certificates. This method requires individual death certificates with multiple cause-of-death data. This method assumes that for all causes of death that a certifying physician lists as heart failure, he or she is equally likely to omit the underlying cause of death from the death certificate (regardless of whether the underlying cause is known). We performed a preliminary validation of the method. The validation indicated that even if the underlying cause of death is more likely to be omitted for certain demographic groups, the method would work well.

Using a nonparametric method such as matching to correct cause-of-death data has a number of advantages over multinomial logistic regression, which has been used elsewhere [12,13]. First, this method is fast compared to multinomial regression. Second, it does not impose assumptions about the functional form and therefore, unlike regression, is unaffected if those assumptions are wrong. Matching is equivalent to a fully saturated multinomial model, including all pairwise and higher order interactions, but without assuming that treatment effects are constant. Using a matching algorithm results in an algorithm that is insensitive to analysts' choices about whether to include interactions and higher order terms [20]. Third, we do not assume parameter constancy (that all of the predictor variables mean the same thing for all observations). This assumption may not hold if the variation in the parameters is related to the relatively small number of available covariates. If this is the case, the results would very likely be biased. Fourth, logistic regression can be biased if its crucial "independence of irrelevant alternatives" assumption is violated; coarsened exact matching is not biased whether or not this assumption holds. An implication of this is that the outcome categories need not be broad and distinct when coarsened exact matching is used. Finally, an important related advantage of matching is that it does not require the analyst to select the underlying causes of death to which ill-defined deaths are reassigned. In fact, it identifies the causes of death with which specific ill-defined causes of death are associated. For example, it is implausible that heart failure is in the causal chain for cancers, yet certifying physicians frequently list heart failure and cancers together on the death certificate. This method identifies that association and redistributes heart failure deaths accordingly. A multinomial regression using the match variables in the base case and the outcome categories identified using matching yields quite similar results to the matching algorithm -- but arriving at the model using multinomial regression alone would have required more stringent assumptions, as well as fitting a larger number of models, and therefore more time and computational resources.

The method described here could be applied to other intermediate cause-of-death codes that are frequently recorded on death certificates, such as septicemia (ICD-10 A40-A41). It could also be applied to underlying causes of death that are used inconsistently for different demographic groups, such as diabetes [13], or liver cirrhosis and liver cancer. Death records for a demographic group for whom certification is expected to be of poor quality can be matched to records for a reference demographic group for whom certification is of high quality.

This method has several limitations. First, the validation presented did not test the assumption that the probability of omitting the underlying cause of death is equal across causes for which heart failure is listed. To validate that assumption, a review of medical records and/or autopsies of a random sample of deaths certified to heart failure, and a tally of the revised underlying causes of death, would be needed. Second, death records can only be matched on recorded covariates. The results could be improved by measuring and including additional covariates (such as additional indicators of socioeconomic status or additional signs and symptoms not recorded on the death certificate) and assessing the results. Finally, the redistribution algorithm may not be transferable to other countries. Even if the assumptions of how heart failure is used hold true within each of the three countries analyzed, physician culture surrounding the use of heart failure is likely to vary from country to country. For example, 18% of recorded deaths were certified to heart failure in Egypt in 2007 [21]; we suspect that physicians commonly use heart failure when the cause of death is unknown. The corresponding correct underlying causes of these deaths likely represent a broader range of underlying causes than in the US, Mexico, or Brazil.

A challenge when interpreting cause-of-death statistics is distinguishing between true epidemiological differences across demographic groups and variations in quality of cause-of-death assignment. For example, there is a clear association both across and within the countries studied between use of hypertensive heart disease as an underlying cause of death (and therefore, redistribution of heart failure deaths to hypertensive heart disease) and indicators of low socioeconomic status. Individuals with low socioeconomic status are less likely to have diagnosed and controlled their hypertension, and therefore would be more likely to die from hypertensive heart disease. However, it is also plausible to argue that hypertensive heart disease is overused as an underlying cause of death, and that overuse is higher among groups with inferior access to health care. Likewise, the high proportion of heart failure deaths reassigned to diabetes in Mexico may represent a true epidemiological difference, or merely physician practice surrounding the certification of deaths to diabetes. As with a complete validation of this method, resolving such doubts would require review of medical records and/or autopsies of a random sample of hypertensive heart disease deaths.

ICD rules for designating the underlying cause of death represent a categorical system of classification; that is, each death is assigned one and only one cause. Categorical classification has the advantage that deaths from each disease sum to the total number of deaths [22]. However, in some cases, including with many heart failure deaths, several diseases contribute to a given death, and the death may have been delayed by removing any one of the disease factors. This can make categorical attribution of the death to a single cause somewhat arbitrary [22]. Relatedly, policymakers may be interested in the entire chain of risks and diseases that lead to a given death so that they can estimate the effect of intervening early in the causal chain (i.e., promoting physical activity to reduce hypertension) or at a later stage (i.e., improving management of patients with heart failure). Nevertheless, there is currently no consensus on an alternate (counterfactual) method for classifying deaths. An important first step is to collect multiple cause-of-death information, and make these data available for analysis, as done by the US. This allows researchers to assign deaths according to their specific research goals. We encourage other national statistical offices to collect and disseminate multiple cause-of-death data to allow for this type of research.

## Conclusions

Reassigning ill-defined deaths to plausible underlying causes of death reduces bias in cause-specific mortality rates and increases comparability of mortality statistics over time and across demographic groups. In this paper, we suggest that coarsened exact matching be used to identify causes of death to which deaths should be redistributed and to derive situation-specific redistribution algorithms. We performed a preliminary validation of the method, and suggest that it be validated with a review of medical records or autopsies of deaths certified to heart failure.

## Abbreviations

ARE: average relative error; COPD: chronic obstructive pulmonary disease; IHD: ischemic heart disease; MCD: multiple cause of death; UCD: underlying cause of death

## Competing interests

The authors declare that they have no competing interests.

## Authors' contributions

GK developed the methods described in this paper. GK and GS tailored the methods for this application. GS carried out the calculations and drafted the manuscript. KS conceived of and coordinated the study. All authors helped to write the manuscript, and have read and approved the final manuscript.

## Disclaimer

The view presented in this paper does not necessarily represent the view of the World Health Organization.

## Supplementary Material

Additional file 1 Additional tables, which contains Tables S1-S4. Click here for file
