# Supplementary material for: Deaths from heart failure: using coarsened exact matching to correct cause-of-death statistics
Source: Popul Health Metr. 2010 Apr 13;8:6. doi: 10.1186/1478-7954-8-6 (PMC2873307; doi:10.1186/1478-7954-8-6)
Supplement: Additional file 1 — Additional tables, which contains Tables S1-S4. [file 1478-7954-8-6-S1.PDF]

Deaths from heart failure: Using coarsened exact matching to correct cause of death statistics

Stevens GA, King G, and Shibuya K.

Table S1. Redistribution algorithm for the US, disaggregated by demographic groups. HS: high school.

	Sex		Death location		Education			Race			Hispanic status		
	Males	Females	In a hospital or clinic	Elsewhere	Less than HS	At least HS	At least college	White	Black	Other	Hispanic	Non-hispanic	Overall
Lower respiratory infections	1%	2%	2%	1%	2%	1%	1%	1%	1%	2%	1%	1%	1%
Diabetes	6%	6%	6%	6%	6%	6%	5%	6%	9%	8%	11%	6%	6%
Cancers	5%	4%	4%	5%	4%	4%	4%	4%	6%	4%	4%	4%	4%
Ischemic heart disease	55%	51%	53%	51%	53%	53%	54%	54%	41%	50%	51%	53%	53%
Cerebrovascular disease	1%	2%	2%	1%	2%	2%	2%	2%	3%	2%	2%	2%	2%
Hypertensive heart disease	5%	8%	6%	8%	7%	7%	7%	6%	14%	8%	8%	7%	7%
Cardiomyopathy	6%	4%	4%	5%	4%	5%	5%	4%	8%	5%	5%	4%	4%
Other cardiovascular diseases	8%	12%	10%	10%	9%	11%	12%	11%	7%	9%	7%	10%	10%
Chronic obstructive pulmonary disease (COPD)	5%	4%	5%	5%	5%	5%	3%	5%	3%	4%	3%	5%	5%
Digestive diseases	1%	1%	1%	1%	1%	1%	1%	1%	1%	1%	2%	1%	1%
Other diseases	6%	7%	7%	6%	7%	6%	7%	6%	8%	6%	7%	7%	7%

Table S2. Redistribution algorithm generated for Mexico, disaggregated by demographic groups.

	Sex		Place of death		Education			Health Insurance			Overall
Matched cause	Males	Females	Other	Hospital/ clinic	Up to Primary	At least Primary	At least Secondary	IMSS	Other private/public	Seguro popular/none	
Lower respiratory infections	3%	3%	2%	3%	3%	2%	2%	2%	2%	3%	3%
Diabetes	11%	15%	11%	18%	12%	18%	14%	17%	17%	11%	13%
Cancers	6%	5%	6%	5%	5%	7%	10%	7%	8%	5%	6%
Ischemic heart disease	27%	26%	27%	24%	26%	26%	30%	28%	26%	25%	26%
Cerebrovascular disease	3%	3%	3%	2%	3%	2%	2%	2%	3%	3%	3%
Hypertensive heart disease	14%	18%	17%	13%	17%	15%	12%	15%	15%	17%	16%
Cardiomyopathy	1%	1%	0%	2%	1%	1%	2%	1%	1%	1%	1%
Other cardiovascular diseases	8%	8%	8%	8%	8%	7%	9%	7%	7%	9%	8%
Chronic obstructive pulmonary disease (COPD)	13%	10%	11%	12%	12%	8%	8%	11%	11%	12%	11%
Digestive diseases	6%	3%	4%	5%	4%	4%	4%	3%	3%	4%	4%
Other diseases	10%	9%	10%	8%	9%	9%	8%	7%	7%	11%	9%

Table S3. Redistribution algorithm generated for Brazil, disaggregated by demographic groups.

	Sex		Place of death		Education			
Matched cause	Males	Females	Other	Hospital/clinic	None	Up to 7 years	More than 7 years	Overall
Lower respiratory infections	3%	3%	1%	3%	3%	3%	2%	3%
Diabetes	7%	11%	8%	9%	9%	9%	9%	9%
Cancers	4%	3%	3%	3%	3%	3%	4%	3%
Ischemic heart disease	23%	21%	25%	21%	18%	23%	31%	22%
Cerebrovascular disease	4%	4%	5%	4%	5%	4%	3%	4%
Hypertensive heart disease	21%	24%	30%	20%	27%	21%	15%	23%
Cardiomyopathy	10%	9%	7%	10%	7%	10%	11%	9%
Other cardiovascular diseases	5%	6%	3%	6%	3%	6%	9%	5%
Chronic obstructive pulmonary disease (COPD)	10%	8%	8%	9%	10%	9%	5%	9%
Digestive diseases	3%	2%	1%	2%	2%	2%	2%	2%
Other diseases	11%	10%	9%	11%	11%	10%	9%	11%

Table S4. Redistribution algorithm for specified heart failure (congestive and left ventricular, I50.0 and I50.1) and for unspecified heart failure (I50.9), base case. HF: Heart Failure.

	USA		Mexico		Brazil	
	Specified HF (I50.0 and I50.1)	Unspecified HF (I50.9)	Specified HF (I50.0 and I50.1)	Unspecified HF (I50.9)	Specified HF (I50.0 and I50.1)	Unspecified HF (I50.9)
Distribution of heart failure deaths	93%	7%	47%	53%	62%	38%
Lower respiratory infections	1%	2%	2%	3%	3%	3%
Diabetes	6%	5%	14%	12%	9%	9%
Cancers	4%	9%	4%	6%	2%	4%
Ischemic heart disease	53%	46%	25%	27%	20%	24%
Cerebrovascular disease	2%	3%	3%	3%	4%	5%
Hypertensive heart disease	7%	6%	17%	16%	25%	20%
Cardiomyopathy	5%	4%	1%	1%	11%	8%
Other cardiovascular diseases	10%	9%	9%	8%	5%	6%
Chronic obstructive pulmonary disease (COPD)	5%	4%	13%	10%	9%	8%
Digestive diseases	1%	3%	4%	4%	2%	2%
Other diseases	6%	10%	8%	10%	10%	11%
